# Correlation between antimicrobial resistance, biofilm formation, and virulence determinants in uropathogenic *Escherichia coli* from Egyptian hospital

**DOI:** 10.1186/s12941-024-00679-2

**Published:** 2024-02-24

**Authors:** Sara A. Alshaikh, Tarek El-banna, Fatma Sonbol, Mahmoud H. Farghali

**Affiliations:** Department of Pharmaceutical Microbiology, Faculty of Pharmacy, Tanta University, Tanta, 31511 Egypt

**Keywords:** Uropathogenic *Escherichia coli*, Antimicrobial resistance, Extended spectrum β-lactamases, Virulence factors, Biofilm formation, Phylogenetic group, ERIC-PCR

## Abstract

**Background:**

Uropathogenic *Escherichia coli* (UPEC) is the main etiological agent behind community-acquired and hospital-acquired urinary tract infections (UTIs), which are among the most prevalent human infections. The management of UPEC infections is becoming increasingly difficult owing to multi-drug resistance, biofilm formation, and the possession of an extensive virulence arsenal. This study aims to characterize UPEC isolates in Tanta, Egypt, with regard to their antimicrobial resistance, phylogenetic profile, biofilm formation, and virulence, as well as the potential associations among these factors.

**Methods:**

One hundred UPEC isolates were obtained from UTI patients in Tanta, Egypt. Antimicrobial susceptibility was assessed using the Kirby-Bauer method. Extended-spectrum β-lactamases (ESBLs) production was screened using the double disk synergy test and confirmed with PCR. Biofilm formation was evaluated using the microtiter-plate assay and microscopy-based techniques. The phylogenetic groups of the isolates were determined. The hemolytic activity, motility, siderophore production, and serum resistance of the isolates were also evaluated. The clonal relatedness of the isolates was assessed using ERIC-PCR.

**Results:**

Isolates displayed elevated resistance to cephalosporins (90–43%), sulfamethoxazole-trimethoprim (63%), and ciprofloxacin (53%). Ninety percent of the isolates were multidrug-resistant (MDR)/ extensively drug-resistant (XDR) and 67% produced ESBLs. Notably, there was an inverse correlation between biofilm formation and antimicrobial resistance, and 31%, 29%, 32%, and 8% of the isolates were strong, moderate, weak, and non-biofilm producers, respectively. Beta-hemolysis, motility, siderophore production, and serum resistance were detected in 64%, 84%, 65%, and 11% of the isolates, respectively. Siderophore production was correlated to resistance to multiple antibiotics, while hemolysis was more prevalent in susceptible isolates and associated with stronger biofilms. Phylogroups B2 and D predominated, with lower resistance and stronger biofilms in group B2. ERIC-PCR revealed considerable diversity among the isolates.

**Conclusion:**

This research highlights the dissemination of resistance in UPEC in Tanta, Egypt. The evident correlation between biofilm and resistance suggests a resistance cost on bacterial cells; and that isolates with lower resistance may rely on biofilms to enhance their survival. This emphasizes the importance of considering biofilm formation ability during the treatment of UPEC infections to avoid therapeutic failure and/or infection recurrence.

**Supplementary Information:**

The online version contains supplementary material available at 10.1186/s12941-024-00679-2.

## Background

Urinary tract infections (UTIs) are among the most prevalent infections in humans in all age groups [1], with an estimated 150 million infections every year around the world [2]. Uropathogenic *Escherichia coli* (UPEC) is by far the most frequently encountered cause of UTIs, causing up to 95% of community-acquired UTIs and more than 50% of all catheter-associated UTIs [1, 3].

Antimicrobial resistance (AMR) has emerged as a serious threat to global health, in the US alone, the incidence of antibiotic-resistant infections surpasses 2.8 million cases, leading to over 35,000 deaths yearly [4]. By the year 2050, it is anticipated that AMR will cause 10 million more deaths [5, 6]. The large-scale dissemination of antimicrobial resistance in uropathogens, including UPEC, is driven by the extensive use of antibiotics in UTI treatment, even for brief periods [7]. Subsequently, the treatment of UTIs has become progressively challenging as a result of the rapid spread of multidrug-resistant (MDR) strains, especially in patients with recurrent UTIs [8]. Recent studies revealed that pathogenic *E. coli* strains are becoming more resistant to a wide range of antibiotic classes, including β-lactams, tetracyclines, fluoroquinolones, trimethoprim-sulfamethoxazole, and aminoglycosides [7, 9–11]. As resistance patterns vary widely between different regions, it is crucial to identify the local bacterial AMR profile to prevent treatment failure and lower the risk of complications, which is especially relevant in cases of infections brought on by MDR strains [12, 13].

Biofilm formation on biological surfaces is an attribute that prevents the elimination of UPEC during antibiotic therapy. Biofilms are organized 3D multicellular aggregates that can attach to biological or abiotic surfaces and are encased in an extracellular polymeric substance (EPS) that is self-produced [14]. UPEC cells can create biofilms on the surface of catheters, inside bladder epithelial cells, and on bladder walls [2, 15, 16]. These biofilms display unique qualities that are not present in free-living cells, including high resilience against extrinsic stressors, such as pH changes, nutrient deficiencies, host immunity, as well as the penetration of antimicrobial agents [3]. Extensive research on the relationship between antimicrobial resistance and biofilm formation capacity has produced contradictory findings. It was reported that biofilm formation enhances bacterial resistance by several mechanisms, such as reduced antimicrobial diffusion and delayed growth rates [13, 17]. Conversely, other studies suggested that antimicrobial resistance bears a considerable fitness cost on bacterial cells, which may reduce their biofilm formation ability. Biofilms may serve as an independent mechanism for bacterial resistance, primarily employed by susceptible isolates as a survival strategy [18–21]. On the other hand, a lack of a discernible link between antimicrobial resistance and biofilm formation capacity was also reported [22, 23]. This is further complicated by the fact that antimicrobial susceptibility testing is conventionally performed on planktonic cells with no regard to their biofilm formation abilities. A major difference in the sensitivity between biofilm-embedded cells and cells in the planktonic state belonging to the same strain has been reported [20, 24]. Together, these observations stress the importance of considering biofilm formation ability as a vital bacterial determinant in the planning of UTI treatment [25].

UPEC strains display a variety of virulence determinants that are essential to their ability to multiply in the urinary tract, attach to host cells, invade the urothelium, adapt to various host-related conditions, and evade the immune system [26]. Flagellar motility enables the bacterium to ascend the urethra and colonize the bladder [27]. Several toxins, such as hemolysin, are secreted during the infection to cause tissue damage and ensure nutrient release. Also, iron is sequestered from host tissues with the aid of iron-acquisition molecules called siderophores to ensure survival in iron-deficient environments such as the urinary tract [28]. Serum resistance is another important virulence trait that enhances the ability of pathogenic *E. coli* strains to evade the immune system of the host and increases their urosepsis potential [29, 30].

Management of UTIs requires prompt administration of the proper antibiotics. However, effective treatment should be based on evidence from regional antimicrobial susceptibility patterns, awareness of the biofilm formation capability and virulence profile, and a thorough comprehension of the association between these factors [13, 30, 31]. Therefore, this study aims to explore the phylogenetic grouping, antimicrobial resistance, biofilm formation capacity, and virulence profile of clinical UPEC isolates collected from UTI patients in Tanta, the capital of the Gharbia governorate in Egypt, and to evaluate the relationships between these factors, which may help establish effective measures for the future prevention and management of UTIs in Egypt.

## Methods

### Isolation and identification of bacterial isolates

Over the period of November 2021 to May 2022, a total of three hundred twenty-seven clinical urine specimens were obtained from midstream urine and catheter-aspirated urine of patients diagnosed with UTIs by a urologist and admitted to the Urology and Nephrology department at Tanta University Hospital, Egypt.

All the specimens were aseptically collected by the Tanta University Hospital personnel before the initiation of antimicrobial therapy. A bacterial count of ≥ 10^5^ CFU/mL was used to define positive urine cultures [32]. The isolated bacteria were initially identified by examination of the colonial morphology on various culture media, including blood agar, Cysteine Lactose Electrolyte Deficient (CLED) agar (Oxoid, USA), MacConkey agar (Oxoid, USA), and HiCrome™ UTI agar (Himedia, India), as well as Gram staining technique. The identification of *E.coli* was further confirmed by a series of standard biochemical tests (methyl red test, KIA test, Vogues Proskauer test, indole test, citrate test, and urease test) [33]. Additionally, the identity of representative isolates of positively identified *E. coli* was confirmed using Matrix-Assisted Laser Desorption/Ionization-Time of Flight Mass Spectrometry (MALDI-TOF\MS) (MALDI Biotyper®, Bruker Daltonics, Germany), which is a reliable technique for bacterial identification with high accuracy [34]. The isolates were then preserved in tryptic soy broth (TSB) supplemented with 20% glycerol at -80 °C for long-term storage.

### Antimicrobial susceptibility testing (AST)

The Clinical and Laboratory Standards Institute (CLSI 2020) guidelines were followed for testing the antimicrobial susceptibility of the isolates. As for tigecycline, the European Committee on Antimicrobial Susceptibility Testing (EUCAST 2022) guidelines were used. The susceptibility of the isolates to twenty-two antimicrobial agents belonging to seventeen categories was assessed. The Kirby-Bauer disc diffusion method was used for the following agents: gentamicin (GEN, 10 μg), amikacin (AK, 30 μg), imipenem (IPM, 10 μg), meropenem (MRP, 10 μg), cefazolin (CZ, 30 μg), cefuroxime (CXM, 30 μg), cefoxitin (FOX, 30 μg), cefotaxime (CTX, 30 μg), ceftazidime (CAZ, 30 μg), cefepime (CPM, 30 μg), ampicillin (AMP, 10 μg), aztreonam (AT, 30 μg), amoxicillin-clavulanic acid (AMC, 10–20 μg), piperacillin-tazobactam (TZP, 100-10 μg), fosfomycin (FO, 200 μg), ciprofloxacin (CIP, 5 μg), trimethoprim-sulfamethoxazole (SXT, 1.25–23.75 μg), chloramphenicol (C, 30 μg), tetracycline (TE, 30 μg), tigecycline (TGC, 15 μg), and nitrofurantoin (NIT, 300 μg) (Oxoid, USA), while susceptibility to colistin (CL) was determined using the broth microdilution method. *E. coli* ATCC 25922 and *Pseudomonas aeruginosa* ATCC 27853 were utilized as reference strains for the quality control of the susceptibility tests. According to Magiorakos et al. [35], isolates that resist ≥ 3 classes of antimicrobials were categorized as MDR, while isolates displaying resistance to all but two or fewer classes were considered XDR. All the antimicrobial groups designated by Magiorakos et al. were used in this study except for the anti-MRSA cephalosporins (ceftaroline). The multiple antibiotic resistance (MAR) indexes were calculated as previously described [36].

### Extended-spectrum β-lactamase (ESBL) production

According to the CLSI guidelines (2020), reduced sensitivity to broad-spectrum cephalosporin antibiotics suggests a possible ESBL-producing strain. The double-disk synergy test (DDST) was performed to screen those isolates for ESBL production, according to a previously described procedure [37]. In brief, isolates were plated on a Mueller Hinton agar, an amoxicillin-clavulanic acid (10–20 μg) disc was then centrally placed on the inoculated plate, surrounded by discs of broad-spectrum cephalosporins, placed 30 mm apart. After incubation, the presence of a synergistic enhancement of the zone of inhibition of any disc towards the amoxicillin-clavulanic acid disc indicated a positive result*.* The reference strain* E. coli* ATCC 25922 was used as a negative control.

### Biofilm formation assay

The biofilm formation capacity of the isolates was evaluated using the 96-well microtiter plate assay as previously described [17]. In short, bacterial suspensions in Luria Bertani (LB) broth were incubated at 37 °C for 18–24 h with shaking. Afterward, the suspensions were diluted 1: 100 in M63 medium containing 0.25% glucose to a final volume of 200 µL. Subsequently, the inoculated microtiter plates were incubated without shaking at 30 °C for 48 h. Following the removal of the culture, sterile phosphate-buffered saline (PBS) was used to wash the wells. After drying at 65 °C, biofilms were stained for 10 min with 2% crystal violet (CV), washed with PBS, and then dried at 65 °C. The adherent CV was then dissolved in 33% glacial acetic acid, and the optical density at 580 nm (OD_580_) was measured using a microplate reader (Sunrise™, TECAN, Switzerland) to determine the biofilm formation capacity. The uninoculated medium served as a negative control. The assay was carried out in triplicate. As previously described [38], the cutoff value (ODc) was calculated as three standard deviation units above the average absorbance of the sterile media. The isolates were subsequently classified into non-biofilm-producing isolates (OD ≤ ODc), weak biofilm-producing isolates (ODc ≥ OD ≤ 2ODc), moderate biofilm-producing isolates (2ODc ≥ OD ≤ 4ODc), and strong biofilm producing isolates (OD ≥ 4ODc).

### Microscopical visualization of biofilms

Representative isolates with strong, moderate, and weak biofilm formation were visualized using light microscopy (LM), scanning electron microscopy (SEM), and confocal laser scanning microscopy (CLSM) for the comparison between their biofilm production abilities and the architecture of the mature biofilms. The LM examination was conducted as previously described [39]. Briefly, biofilms were formed as described above in 6-well plates, with each well containing one 1 × 1 cm sterile glass slide. After incubation, the glass slides were carefully taken out using sterile forceps and were washed with PBS to eliminate the nonadherent cells. After staining the slides with 0.2% CV for 5 min, the slides were washed and allowed to dry then examined using a compound brightfield light microscope at 100 × and 400 × magnifications (Labomed, California, USA). The SEM examination was performed as previously described [40]. Initially, the biofilms were formed on glass slides as described above. Biofilms were subsequently fixed with 2.5% glutaraldehyde for 30 min at 37 °C. The slides were washed thrice using PBS and dehydrated using ethanol, then sputter coated with gold. Finally, the scanning electron microscope (S-34002N SEM, Hitachi^®^, Tokyo, Japan) was used to examine the biofilms.

The architecture of biofilms was further assessed using CLSM, as reported by Karunanidhi et al. [41]. Biofilms were formed in an 8-well µ-Slide (ibidi, Martinsried, Germany). After 48 h of incubation, the chamber was washed with PBS to eliminate the planktonic cells, and the biofilm mass was stained by acridine orange (Invitrogen) which produces green fluorescence upon exposure to the laser beam. The stained biofilms were subsequently visualized under a confocal laser scanning microscope (DMi8; Leica Microsystem).

### Phenotypic determination of virulence factors

#### Hemolytic activity

The hemolytic capacity of the tested isolates was qualitatively and quantitatively evaluated using previously described methods [11]. For qualitative purposes, screening for hemolysis was performed using 5% blood agar plates. For quantitative hemolysis determination, the tube assay method was performed as follows: isolates were incubated in TSB broth for 48 h at 37 °C, followed by centrifugation for 10 min at 10,000 rpm. An equal volume of the supernatant and RBC suspension (2%) were mixed before incubation for 2 h at 37 °C, then the mixture was centrifuged at 4 °C to precipitate RBCs. Hemoglobin release in the collected supernatant was detected by measuring the OD at 540 nm. The hemolytic activity (%) was then calculated using the following equation:

$$\mathrm{Hemolytic \,\,activity }(\mathrm{\%}) = \frac{{\text{x}}-{\text{b}}}{t-b}\times 100$$

where b is the OD_540_ of the negative control composed of sterile medium mixed with the RBCs suspension, and t is the OD_540_ of the positive control prepared by mixing the RBCs suspension with sodium dodecyl sulfate (0.1%). Additionally, x represents the OD_540_ value of the sample analyzed. A Triplicate of the experiment was performed.

### Serum survival assay

The resistance of the isolates to human serum was determined as previously reported [42]. Blood was collected from healthy donors in plain serum collection tubes. After allowing the blood to clot at room temperature for 30 min, the clot was removed by centrifugation at 3000 rpm for 10 min in a refrigerated centrifuge (4 °C) (Sigma 2-16KL, Germany), and the serum was immediately separated. Overnight cultures of the isolates in LB broth were centrifuged, washed, and resuspended in sterile PBS and adjusted to OD_600_ equal to 0.1. A volume of 100 µL of the adjusted inoculum was combined with an equal amount of the serum, and the mixture was then incubated for 2 h at 37 °C. At 0 h and after 2 h of incubation, aliquots of ten microliters were plated onto nutrient agar after serial dilution, and the surviving bacterial count was determined after the agar plates were incubated for 24 h at 37 °C. Bacterial survival was calculated as a proportion of the initial count at 0 h. The isolate was considered serum-sensitive when the count declined to ≤ 1%. When the remaining count was ≥ 90% of the initial count the isolate was considered serum-resistant, a count between 1 and 90% of the initial count indicated intermediate resistance.

### Siderophore production assay

As previously described [43, 44], the universal Chrom Azurol S (CAS) assay was used to examine the bacterial isolates' capacity to produce siderophores. One hundred mL of the Chrome Azurol S- hexadecyltrimethylammonium bromide (CAS-HDTMA) solution was mixed with 900 mL of sterile LB agar medium to prepare the CAS agar plates. The plates were inoculated with the isolates and incubated for 5 days at 28 °C. After incubation, the plates were examined for the production of an orange zone surrounding the bacterial growth.

### Motility assay

The motility of the isolate was evaluated as previously described [45]. The isolates were stab-inoculated into semisolid agar tubes containing 0.4% agar and then incubated for 48 h at 37 °C. After incubation, the tubes were examined for motility, where motile bacteria spread beyond the inoculation line while non-motile bacteria remained at the stab site.

### Bacterial DNA extraction

The isolates' total DNA was extracted using the boiling lysis technique [46]. Briefly, the overnight culture suspensions of isolates were centrifuged, and the cell pellets were resuspended in 200 µL of nuclease-free water. After being boiled for 10 min at 100 °C, the suspensions were rapidly placed on ice for 5 min, this was followed by centrifugation for 30 s at 13,000 rpm. A NanoDrop One spectrophotometer (Thermo Fisher Scientific, USA) was used to assess the purity and concentration of the DNA yield [47]. The DNA-containing supernatants were then stored in small aliquots at – 20 °C until use.

### Phylogenetic group determination

The phylogenetic classification of the isolates was assigned as per the Clermont revised scheme [48]. This method entails a multiplex PCR reaction for the detection of four genes, namely, *arpA, chuA*, *yjaA*, and TspE4.C, followed by two allele-specific reactions for the detection of phylogroups E and C. The primers used for these reactions are listed in Table 1. Table 2 includes the scheme for the assignment of the phylogenetic groups.

**Table 1:** The primers used for the PCR reactions in this study

Gene	PCR reaction	Primer ID	Primer sequence	Product size (bp)
Phylogenetic group assignment^a^				
*chuA*	Quadruplex reaction	chuA.1b	5′—ATGGTACCGGACGAACCAAC-3′	288
		chuA.2	5′—TGCCGCCAGTACCAAAGACA-3	
*yjaA*		yjaA.1b	5′—CAAACGTGAAGTGTCAGGAG-3′	211
		yjaA.2b	5′—AATGCGTTCCTCAACCTGTG-3	
*TspE4.C*		TspE4C2.1b	5′—CACTATTCGTAAGGTCATCC-3′	152
		TspE4C2.2b	5′—AGTTTATCGCTGCGGGTCGC-3′	
*arpA*		AceK.f	5′—AACGCTATTCGCCAGCTTGC-3′	400
		ArpA1.r	5′—TCTCCCCATACCGTACGCTA-3′	
*arpA*	Group E	ArpAgpE.f	5′—GATTCCATCTTGTCAAAATATGCC-3′	301
		ArpAgpE.r	5′—GAAAAGAAAAAGAATTCCCAAGAG-3′	
*trpA*	Group C	trpAgpC.1	5′—AGTTTTATGCCCAGTGCGAG-3′	219
		trpAgpC.2	5′—TCTGCGCCGGTCACGCCC-3	
*trpA*	Internal control gene in group E and C reactions	trpBA.f	5′—CGGCGATAAAGACATCTTCAC-3′	489
		trpBA.r	5′—GCAACGCGGCCTGGCGGAAG-3′	
Detection of ESBL genes				
*bla*_*SHV*_		bla-SHV.SE	5ʹ—ATGCGTTATATTCGCCTGTG-3′	747
		bla-SHV.AS	5ʹ—TGCTTTGTTATTCGGGCCAA-3′	
*bla*_*TEM*_		TEM-164.SE	5ʹ—TCGCCGCATACACTATTCTCAGAATGA-3′	445
		TEM-164.AS	5ʹ—ACGCTCACCGGCTCCAGATTTAT-3′	
*bla*_*CTX-M*_		CTX-M-U1	5ʹ—ATGTGCAGYACCAGTAARGTKATGGC-3′	593
		CTX-M-U2	5′—TGGGTRAARTARGTSACCAGAAYCAGCGG-3ʹ	

^a^The phylogroup assignment depends on a quadruplex PCR reaction for the detection of the main four genes; *arpA* (400 bp)*, chuA* (288 bp), *yjaA* (211 bp), and TspE4.C (152 bp). Depending on the results of the quadruplex reaction, isolates may be directly assigned to a phylogroup, or follow-up PCR reactions may be required. Follow-up reactions for the detection of the genes *arpA* (301 bp) and *trpA* (219 bp) were performed for the determination of the phylogroups C and E, respectively, where the gene *trpA* (489 bp) is used as an internal control

**Table 2:** The phylogenetic group assignment scheme

Result of the quadruplex reaction				Phylogroup	Follow up reaction
*arpA*	*chuA*	*yjaA*	TspE4.C*2*		
+	−	−	−	A	
+	−	−	+	B1	
−	+	−	−	F	
−	+	+	−	B2	
−	+	+	+	B2	
−	+	−	+	B2	
+	−	+	−	A or C	Group C-specific reaction
+	+	−	−	D or E	Group E-specific reaction
+	+	−	+	D or E	Group E-specific reaction
+	+	+	−	E or Clade I	Group E-specific reaction
−	−	+	−	Clade I or II	
−	(476 bp)^a^	−	−	Clade III, IV, or V	
−	−	−	+	Unknown	
−	−	+	+	Unknown	
+	−	+	+	Unknown	
+	+	+	+	Unknown	
−	−	−	−	Unknown	

^a^In the quadruplex reaction, clades III, IV, or V produce a 467 bp PCR amplicon, which is a consequence of an 11 bp match between the *chuA* gene and the AceK.f primer, in the presence of the chuA.2 primer. These clades do not result in any of the four main amplicons; *arpA (*400 bp)*, chuA* (288 bp), *yjaA (*211 bp), or TspE4.C (152 bp) [48]

### PCR detection of ESBL genes

Sixty-seven isolates showing reduced sensitivity to broad-spectrum cephalosporins were subjected to the detection of important ESBL genes (*bla*_TEM_, *bla*_SHV_, and *bla*_CTX-M_) using multiplex PCR [49]. The thermocycling process conditions involved initial denaturation at 95 °C for 5 min; followed by 30 cycles of denaturation at 94 °C for 30 s, annealing at 56 °C for 30 s, and extension at 72 °C for 1 min; and a final extension step at 72 °C for 10 min. The resulting PCR products were separated by electrophoresis on 1% agarose gel and visualized. The primers used in this reaction are detailed in Table 1.

### Enterobacterial repetitive intergenic consensus (ERIC)-PCR

ERIC-PCR reaction was performed using the primers ERIC-1 (5′- ATGTAAGCTCCTGGGGATTCAC—3ʹ) and ERIC-2 (5′- AAGTAAGTGACTGGGGTGAGCG -3′) as previously described [50, 51]. The reaction mixture was composed of a total volume of 25 μL containing 6 μL of the DNA template, 1 µL of each ERIC primer, 12.5 μL of Dream Taq^™^ Green PCR Master Mix (2x) (Thermo Scientific, US), and 4.5 μL molecular grade water. Thermocycling conditions were initial denaturation at 94 °C for 5 min; followed by 35 cycles of denaturation at 94 °C for 30 s, annealing at 52 °C for 1 min, and extension at 72 °C for 1 min; and a final extension step at 72 °C for 12 min. The PCR amplicons were separated on 1.5% (w/v) agarose gel, and a gel documentation system (Geldoc-it^®^, UVP, England) was employed for visualization. The resulting banding patterns were deciphered using the GelJ^®^ software version 2.00. The banding patterns were then grouped by the unweighted pair group method with an arithmetic average algorithm (UPGMA) using the dice coefficient at 1% tolerance [52]. A cutoff of 80% similarity was selected for group segregation and isolates with 100% similarity were assigned to the same ERIC type.

### Statistical analysis

The Statistical Package for the Social Sciences (SPSS) software, version 27.0 (IBM Corp., Armonk, NY, USA), was used to conduct the statistical analyses. All *P*-values were two-tailed and were regarded as statistically significant at the *P* < 0.05 level. The relationships between categorical variables were assessed using the chi-square test. Spearman's rank correlation was utilized to study the correlation between numerical variables and for intergroup comparisons. The Mann–Whitney U test or the Kruskal–Wallis test was used to compare numerical variables across several groups. Pairwise comparisons and a Bonferroni adjustment for multiple comparisons were conducted after the Kruskal–Wallis test.

## Results

### Bacterial isolation and identification

Over the period of November 2021 to May 2022, a total of 100 UPEC isolates were obtained from the Tanta University Hospital, Egypt. Isolates were subsequently identified based on microscopic examination and standard biochemical tests. Additionally, the identity of representative isolates was confirmed using MALDI-TOF\MS analysis. The patients’ ages ranged between 1 and 78 years (Additional file 1: Table S1). The Mann–Whitney U test showed a significantly higher age mean in male patients (56.7 years) compared to female patients (37.4 years) *(P* < *0.001)*.

### Antimicrobial susceptibility profile

The antimicrobial resistance of the tested isolates was determined towards 22 antibiotics, following the CLSI guidelines (2020) and the EUCAST guidelines (2022). The isolates were categorized as sensitive, intermediate, or resistant. Resistance to CZ was the most common (90%), followed by AMP (88%), CXM (82%), CAZ (65%), SXT (63%), AMC (60%), CTX (60%), TE (57%), CIP (53%), TZP (52%), AT (47%), FOX (47%), CPM (43%), GEN (17%), TGC (13%), C (11%), and NIT (5%). On the other hand, only 0%, 2%, and 2% of the isolates showed resistance to CL, FO, and AK, respectively, while 3% of the isolates displayed resistance to IPM and MRP (Table 3). The MAR index of the isolates ranged between 0 and 0.86 (Additional file 2: Table S2). Moreover, the MAR index increased significantly with age, as revealed by Spearman's correlation *(P* < *0.001)*. Notably, the chi-square test revealed that resistance to CPM and AT was significantly more prevalent in isolates obtained from male patients (*P* < *0.05*). Eighty-eight percent of the isolates were MDR, 2% were XDR, and only 10% were resistant to less than three antimicrobial classes and were categorized as “non-MDR”.

**Table 3:** Overall antimicrobial resistance rates of UPEC isolates against the tested antibiotics, represented as percentages (%)

Antimicrobial category	Antimicrobial agent	Resistance rate (%)^a^		
		R	I	S
*Aminoglycosides*	GEN	17	3	80
	AK	2	3	95
*Carbapenems*	IPM	3	0	97
	MRP	3	1	96
*Non-Extended Spectrum Cephalosporin*	CZ	90	0	10
	CXM	82	4	14
*Extended Spectrum Cephalosporin*	CTX	60	5	35
	CAZ	65	7	28
	CPM^b^	43	15	42
*Fluoroquinolones*	CIP	53	6	41
*Folate Pathway Inhibitors*	SXT	63	3	34
*Glycyclines*	TGC	13	8	79
*Monobactams*	AT	47	9	44
*Penicillins*	AMP	88	4	8
*Penicillin-Beta Lactamase Inhibitor combination*	AMC	60	10	30
*Phenicols*	C	11	1	88
*Phosphonic Acids*	FO	2	0	98
*Tetracyclines*	TE	57	3	40
*Nitrofurans*	NIT	5	3	92
*Cephamycins*	FOX	47	0	53
*Antipseudomonal Penicillin* + *Beta Lactamase Inhibitor combination*	TZP	52	19	29
*Polymyxins*	CL	0	0	100

Gentamicin (GEN), Amikacin (AK), Imipenem (IPM), Meropenem (MRP), Cefazolin (CZ), Cefuroxime (CXM,), Cefoxitin (FOX), Cefotaxime (CTX), Ceftazidime (CAZ), Cefepime (CPM), Ampicillin (AMP), Aztreonam (AT), Amoxicillin-Clavulanic Acid (AMC), Piperacillin-Tazobactam (TZP), Fosfomycin (FO), Ciprofloxacin (CIP), Trimethoprim-Sulfamethoxazole (SXT), Chloramphenicol (C), Tetracycline (TE), Tigecycline (TGC), Nitrofurantoin (NIT), and Colistin (CL). *R* resistant, *I* intermediate resistance, *S* susceptible

^a^Resistance rates (%) are calculated as the proportion of isolates that are R, I, or S to the total number of isolates (*n* = 100), multiplied by 100

^b^For CPM, the CLSI categories include R, SDD (sensitive dose-dependent), and S

Fluoroquinolones are a widely used class of broad-spectrum antimicrobials recommended as first-line treatment in pyelonephritis, complicated UTIs, and prostatitis, as well as being used for UTI prophylaxis [53]. The chi-square test revealed that CIP-resistant isolates had significantly higher resistance rates to the following antibiotics, compared to the CIP-susceptible isolates: GEN* (P* = *0.027),* CXM *(P* = *0.029),* FOX* (P* = *0.036),* CTX *(P* < *0.001)*, CAZ *(P* = *0.002)* CPM *(P* = *0.004)*, SXT *(P* < *0.001),* AMP *(P* = *0.035),* AT *(P* = *0.001),* AMC *(P* = *0.04)*, TZP *(P* = *0.001),* TGC *(P* = *0.013)*, NIT *(P* = *0.037)*, and TE* (P* = *0.014)*.

### The majority of the isolates demonstrated ESBL production

**Fig. 1:**
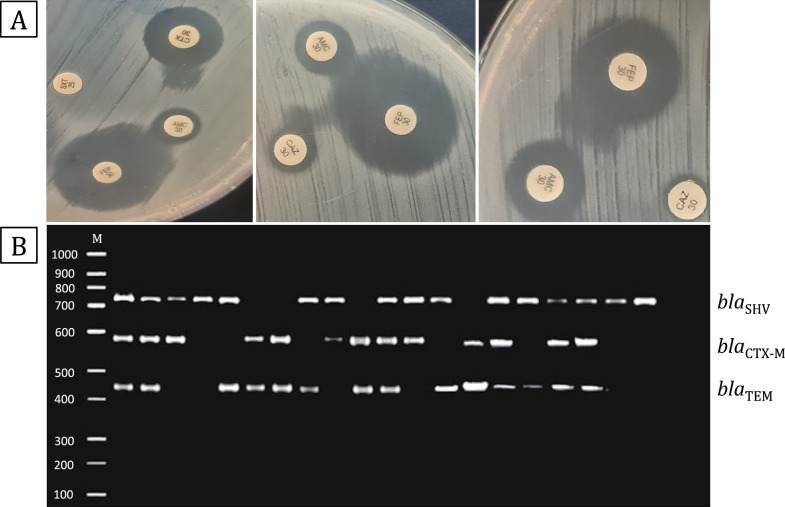
Representative results for the detection of the ESBL production. **A** Representative results for positive ESBL phenotypic detection by the DDST method, illustrate the enhancement of the inhibition zone towards the central AMC disc. **B** Representative results for the detection of ESBL genes using PCR. *bla*_SHV_ (747 bp)*, bla*_TEM_ (445 bp), and *bla*_CTX-M_ (593 bp). M; DNA ladder (100 bp)

Sixty-seven isolates displayed nonsusceptibility to extended-spectrum cephalosporins and were suggested as possible ESBL producers, as recommended by the CLSI guidelines (2020). These isolates were subsequently subjected to the detection of ESBL production using phenotypic and genotypic methods. The phenotypic examination by the DDST showed that only 38 isolates were ESBL producers, displaying an enhancement in the inhibition zone (Fig. 1A).

Multiplex PCR detection of ESBL genes demonstrated that all of the sixty-seven tested isolates possessed at least one of the three tested ESBL genes (Fig. 1B). *bla*_CTX-M_ was the most prevalent ESBL gene, detected in 49 isolates, this was followed by *bla*_TEM_ gene, which was detected in 38 isolates, and *bla*_SHV_ gene which was detected in 36 isolates. As for the ESBL gene combinations detected in the isolates; *bla*_TEM_, *bla*_SHV_, and *bla*_CTX-M_ combination was detected in 13 isolates (19.4%), *bla*_TEM_ and *bla*_CTX-M_ combination was detected in 14 isolates (20.9%), *bla*_SHV_ and *bla*_CTX-M_ combination was detected in 9 isolates (13.4%), while the combination of *bla*_TEM_ and *bla*_SHV_ was detected in 7 isolates (10.4%). On the other hand, 13 isolates (19.4%) possessed only *bla*_CTX-M_, 7 isolates (10.4%) possessed only *bla*_SHV_, while *bla*_TEM_ was found alone in only 4 isolates (5.97%) (Additional file 3: Figure S1). Furthermore, the chi-square test revealed that isolates from male patients were significantly associated with the presence of the ESBL gene *bla*_TEM._ (*P* < *0.05*).

Statistical analysis using the chi-square test revealed that the presence of any ESBL gene had a significant association with resistance to all the β-lactam antibiotics; CZ (*P* < *0.001*), CXM (*P* = *0.003*), FOX (*P* < *0.001*), CTX (*P* < *0.001*), CAZ (*P* < *0.001*), CPM (*P* < *0.001*), AT (*P* < *0.001*), AMP (*P* < *0.001*), TZP (*P* < *0.001*) and AMC (*P* < *0.001*), The presence of ESBL genes was also significantly correlated to the resistance to non-β-lactam antibiotics, namely, GEN (*P* = *0.033*), CIP (*P* = *0.003),* and SXT (*P* = *0.009*).

### Biofilm formation capacity

The biofilm formation capacity of UPEC isolates was evaluated in M63 broth using the 96-well microtiter plate assay which is the gold standard for the estimation of the biofilm formation capacity [54]. Subsequently, the isolates were divided into four categories: strong, moderate, weak, and non-biofilm forming. Only 8% of the isolates did not form a biofilm, while 32% produced weak biofilms, 29% produced moderate biofilms, and 31% produced strong biofilms.

### Microscopic examination of bacterial biofilms using LM, SEM, and CLSM

**Fig. 2:**
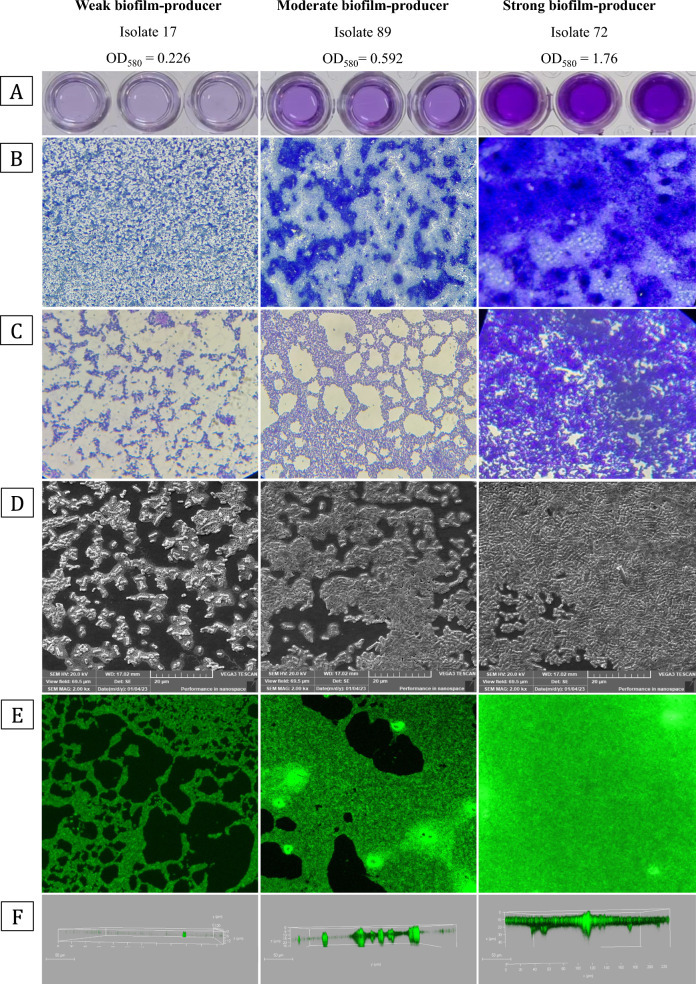
**A** Results of the 96-microtiter plate method for biofilm assay of representative weak, medium, and strong biofilm-producing isolates. **B** and **C** Are microscopic observations of the isolates using a bright field light microscope at a magnification of 100 × and 400 ×. **D** Represents the SEM micrographs of biofilms at 2000×magnification. **E** and **F** Are CLSM micrographs of the acridine orange-stained biofilms in two-dimensional perspective and three-dimensional perspective (side view), respectively. Scale bars represent 20 μm for SEM images and 50 μm for CLSM images

Microscopical examination of biofilm structure was performed on representative isolates of the strong, moderate, and weak biofilm formation categories. The LM examination demonstrated the difference in biomass at the 100 × and 400 × magnifications. Stronger biofilm formation was correlated to a larger amount of CV stain bound to the glass slides, indicating a larger biofilm matrix. According to the SEM analysis, strong biofilm producers showed thick layers of cells, while medium biofilm producers displayed rough biofilms with cell clumps, and weak biofilm producers had small sparse cell clusters. The biofilm architecture was also investigated by CLSM, which revealed a noticeable difference in the two-dimensional and three-dimensional biofilm structure among the isolates. Weak biofilm-producing isolates revealed relatively under-developed biofilms containing a few cell aggregates that were small and scattered. In comparison, medium biofilm producers formed uneven biofilms containing multiple small clusters that were of variable thickness. Strong biofilm producers, on the other hand, showed well-established biofilms with high variability in the biofilm structure and had a higher density of cell clusters (Fig. 2).

### Biofilm formation is inversely correlated to antibiotic resistance

**Fig. 3:**
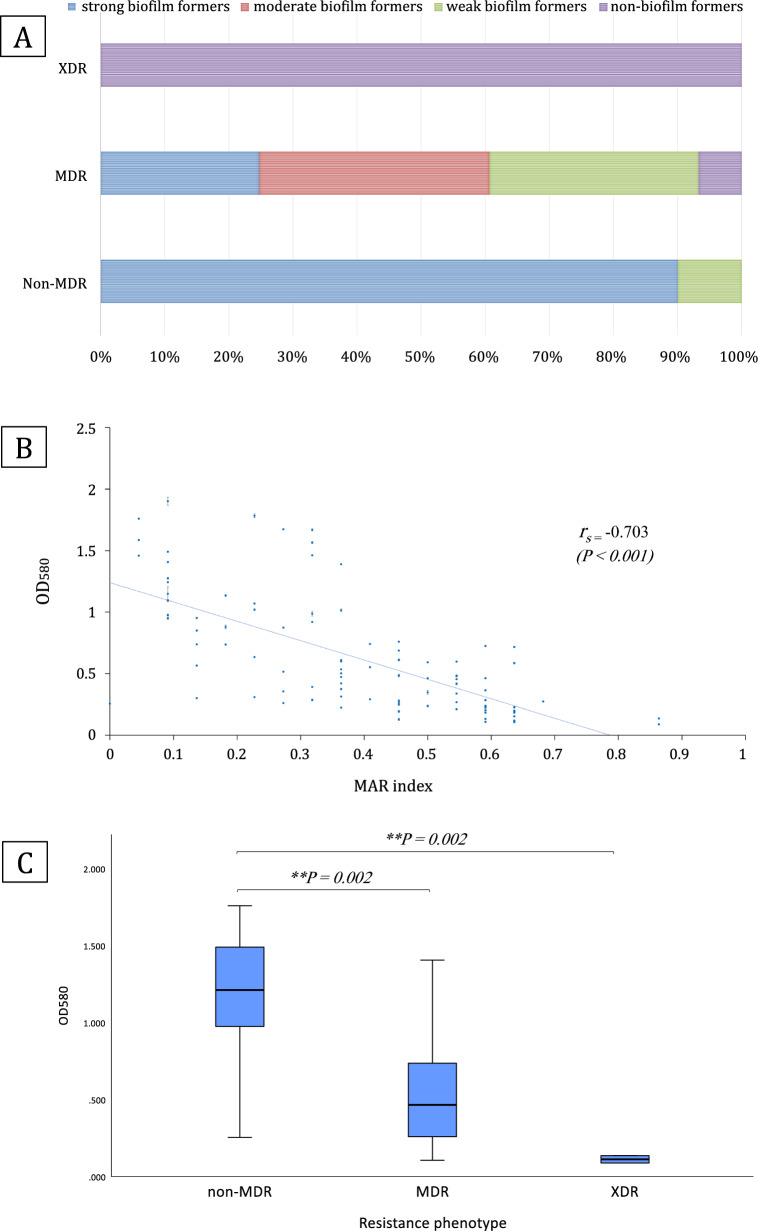
**A** Proportions of biofilm formation categories among different resistance phenotypes. **B** A scatter plot of the MAR index and OD_580_ of each tested isolate indicates the inverse correlation between bacterial resistance and biofilm formation, with a Spearman's correlation coefficient (*r*_*s*_) of -0.703 (*P* < *0.001)*. **C** Biofilm formation capacity among different resistance phenotypes, the Kruskal–Wallis test shows significant differences (*P* < *0.001)*. Subsequent pairwise comparisons reveal that non-MDR isolates had significantly higher OD_580_ compared to MDR isolates (*P* = *0.002*) or XDR isolates (*P* = *0.002*). OD_580_ is the optical density at 580 nm representing the biofilm density

To reveal whether resistance to antibiotics and biofilm formation are correlated, we evaluated the distribution of resistance phenotypes in relation to biofilm formation categories. Our findings revealed that out of 31 isolates, categorized as strong biofilm formers, only 9 isolates (29%) were non-MDR, while the majority (71%) were MDR isolates. Of the weak biofilm formers (32 isolates), one isolate (3.1%) was non-MDR and 31 isolates (96.9%) were MDR. The isolates that tested negative for biofilm formation were all MDR/XDR (Fig. 3A). This distribution suggested that stronger biofilm formers were less likely to be MDR/XDR and that highly resistant isolates tended to produce weaker biofilms. The chi-square test revealed this association to be statistically significant (*P* < *0.001*).

Next, we found that the isolates’ biofilm formation capacity (OD_580_) had a significant negative correlation with their MAR index (Fig. 3B). Spearman's correlation coefficient (*r*_*s*_) was -0.703 (*P* < *0.001*). The Kruskal–Wallis test also showed that non-MDR isolates had significantly higher OD_580_ values than MDR and XDR isolates (*P* < *0.001*) (Fig. 3C).

**Fig. 4:**
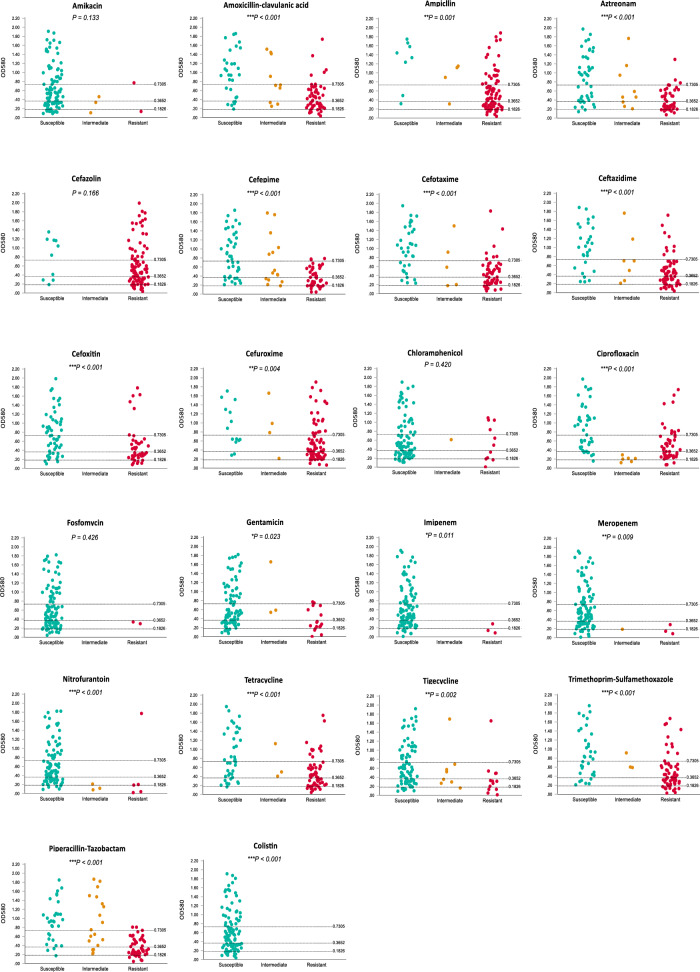
Scatter plots representing the relationship between antibiotic resistance and the biofilm formation ability. Each of the tested 22 antibiotics is included, and the dotted line represents the cutoff for the biofilm formation categories: non-biofilm-forming strains (OD ≤ 0.1826), weak biofilm-forming strains (0.1826 ≥ OD ≤ 0.3652), moderate biofilm-forming strains (0.3652 ≥ OD ≤ 0.7305), and strong biofilm forming strains (OD ≥ 0.7305). Statistical significance at the *P* < *0.05*, *P* < *0.01*, and *P* < *0.001* levels are indicated by the symbols *, **, and ***, respectively

Finally, we statistically analyzed the biofilm formation capacity of the isolates with respect to resistance to each of the tested antibiotics to investigate whether biofilm formation is related to resistance to any antibiotic(s) in particular. Concerning the following 17 antibiotics: GEN, MRP, IPM, CXM, FOX, CAZ, CTX, CPM, CIP, SXT, AMP, AMC, TZP, AT, TGC, NIT, and TE; the Spearman's correlation coefficient showed a significant inverse correlation between resistance and the biofilm formation capacity (*r*_*s*_ = − 0.227 to − 0.614, *P* < *0.05*). On the other hand, no significant correlation was detected for AK, FO, C, and CZ (*r*_*s*_ = − 0.08 to − 0.151, *P* > *0.05*) (Fig. 4). For CL, all the isolates were sensitive and statistical significance could not be investigated.

### The presence of ESBL genes was significantly associated with weaker biofilms

**Fig. 5:**
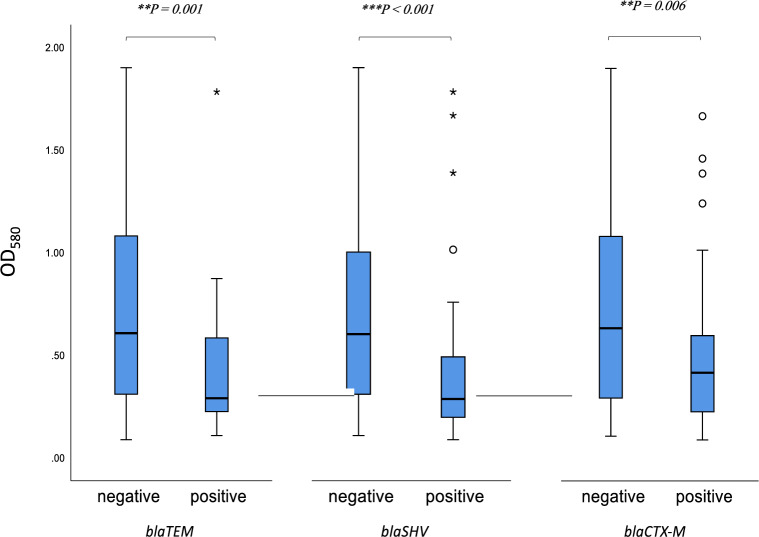
Biofilm formation capacity in relation to the presence of different ESBL genes. The presence of each of the ESBL genes was correlated to significantly lower OD_580_ (*P* < *0.001*)

The chi-square analysis showed that isolates possessing any of the ESBL genes were significantly associated with weaker biofilm formation capacity (*P* < *0.001*). Also, the Mann–Whitney U test revealed that isolates possessing any of the studied ESBL genes had significantly lower average OD_580_ values compared to ESBL-negative isolates (Fig. 5).

### Phenotypic determination of virulence profile

#### Hemolysis assay

The hemolysis assay revealed that 64 isolates out of the 100 tested isolates had hemolytic activity (beta-hemolysis), while the remaining 36 isolates were non-hemolytic (Additional file 4: Figure S2). Notably, both XDR isolates were hemolytic. Statistical analysis by the chi-square test revealed that the beta-hemolytic isolates had a significantly lower prevalence of ESBL production *(P* = *0.001)*, and had significantly higher susceptibility rates to several types of antibiotics, including CIP *(P* = *0.002)*, GEN *(P* = *0.003)*, CTX *(P* < *0.001)*, CAZ *(P* = *0.003)*, CPM *(P* < *0.001)*, AT *(P* = *0.002)*, and TZP *(P* = *0.01)*. The chi-square test also unveiled a significant association between hemolysis and the ability to form biofilms *(P* = *0.021*).

### Serum survival assay

The serum survival assay revealed that 11 isolates were resistant to serum, 60 isolates were serum-sensitive, and 29 isolates had intermediate serum resistance. The Spearman's rank test revealed that serum resistance was directly correlated to the extent of hemolysis (*r*_*s*_ = 0.248, *P* = *0.013*). Both the XDR isolates were serum-sensitive. The chi-square test revealed that serum resistance had no significant association with the ability of the isolates to form biofilms.

### Siderophore production

Siderophore production ability was screened using the CAS agar plates. Siderophore production was indicated by the detection of a yellow zone around the bacterial growth, resulting from the chelation of iron and the release of the free dye [44]. The test revealed that 65 isolates out of the tested 100 isolates were siderophore producers (Additional file 4: Figure S2). The chi-square test revealed that siderophore-producing isolates had significantly higher resistance rates to the following antibiotics: CZ (*P* < *0.001*), CXM (*P* < *0.001*)*,* FOX *(P* = *0.032),* CTX (*P* < *0.001*), CAZ (*P* < *0.001*), CPM (*P* < *0.001*), AT (*P* < *0.001*), AMP (*P* = *0.004*), AMC (*P* = *0.007*), TZP (*P* < *0.001*), and CIP (*P* < *0.001*)*.* Siderophore production was significantly associated with the presence of any of the ESBL genes (*P* < *0.001*).

### Motility test

The motility of the isolates was evaluated using soft motility agar. Eighty-four isolates out of the tested 100 isolates were motile. Both XDR isolates were motile. Motile isolates were found to correlate significantly with the presence of the *bla*_TEM_ gene, compared to non-motile strains *(P* < *0.05)*. The chi-square test revealed that there was no association between biofilm formation and motility.

### Phylogenetic group assignment

The modified scheme devised by Clermont et al. (2013) was used to carry out the phylogenetic classification, the scheme uses a quadruplex PCR reaction for the detection of four essential genes: *chuA, arpA,* TspE4.C2*, and yjaA* (Additional file 5: Figure S3). This modified scheme utilizes enhanced primers to reduce primer mismatches, it also entails follow-up reactions to distinguish previously undistinguishable phylogroups C, E, F, and clade I (Additional file 6: Figure S4). This method categorizes *E. coli* into groups A, B1, B2, C, D, E, and F, as well as cryptic clade I [48].

We found that phylogenetic group B2 was the most prevalent group (37%), followed by phylogenetic group D (25%), phylogenetic group E (11%), phylogenetic group B1 (7%), clade I (6%), and phylogenetic group F (5%), whereas 9% of the isolates were unidentified.

Phylogroups B1 and E included strains isolated from female patients only, while 79% of the isolates from male patients belonged to phylogroups B2 and D. Moreover, phylogroup B1 had the lowest average age compared to any other phylogroup (9.3 years). In contrast, phylogroup D had the highest average age (57.5 years) (Table 4).

**Table 4:** Distribution of patients' age and sex in different phylogroups

Phylogroup	Mean age (Years)	No. of isolates from female patients	No. of isolates from male patients	Total no. of isolates
D	57.5	17	8	25
F	46.2	4	1	5
Clade I	42.5	5	1	6
B2	39.7	30	7	37
E	31.7	11	0	11
Unknown	25.8	7	2	9
B1	9.3	7	0	7
Total	40.4	81	19	100

A comparison between phylogroups B2 and D using the chi-square test displayed that group D had a higher prevalence of all ESBL genes compared to group B2; *bla*_TEM_ (*P* < *0.001*), *bla*_SHV_ (*P* = *0.003*), and *bla*_CTX-M_ (*P* < *0.001*). Also, phylogroup D members had significantly higher rates of resistance to the following antibiotics: CIP (*P* < *0.001*), AK (*P* = *0.018*), TE (*P* < *0.001*), AMP (*P* = *0.018*), AMC (*P* < *0.001*), FOX (*P* < *0.001*), CPM (*P* < *0.001*), CXM (*P* = *0.018*), AT (*P* < *0.001*), CTX (*P* < *0.001*), CAZ (*P* < *0.001*), TZP (*P* < *0.001*), and SXT (*P* < *0.001*). The Mann–Whitney U test also revealed that group D members had a significantly higher MAR index (*P* < *0.001*) compared to group B2. On the other hand, the majority of strong biofilm formers (77.4%) were group B2 members (Table 5). The Mann–Whitney U test showed that group B2 possesses significantly higher biofilm OD_580_ (*P* < *0.001*) compared to group D. The chi-square test also revealed that group B2 had a significantly higher prevalence of strong biofilms (*P* < *0.001*). Moreover, the chi-square test showed that group B2 was significantly associated with beta hemolysis (*P* < *0.001*) in comparison to group D. Contrarily, group D had a higher prevalence of siderophore production in comparison to group B2 (*P* < *0.001*).

**Table 5:** Distribution of phylogroups among different biofilm formation categories

Biofilm category	Phylogroup	No. of isolates	Percentage of phylogroup (%)^a^
Non-biofilm producers	D	4	50
(n = 8)	F	2	25
	B2	1	12.5
	Clade I	1	12.5
Weak biofilm- producers	D	14	43.8
(n = 32)	E	6	18.8
	B1	4	12.5
	B2	3	9.4
	F	2	6.3
	Unknown	2	6.3
	Clade I	1	3.1
Moderate biofilm- producers	B2	9	31
(n = 29)	D	6	20.7
	Unknown	6	20.7
	E	4	13.8
	Clade I	3	10.3
	F	1	3.4
Strong biofilm- producers	B2	24	77.4
(n = 31)	B1	3	9.7
	D	1	3.2
	E	1	3.2
	Clade I	1	3.2
	Unknown	1	3.2

^a^Percentages (%) of each phylogroup are calculated as a proportion to the total number of the isolates within each biofilm formation category, multiplied by 100

### Determination of the clonal relatedness using ERIC-PCR

**Fig. 6:**
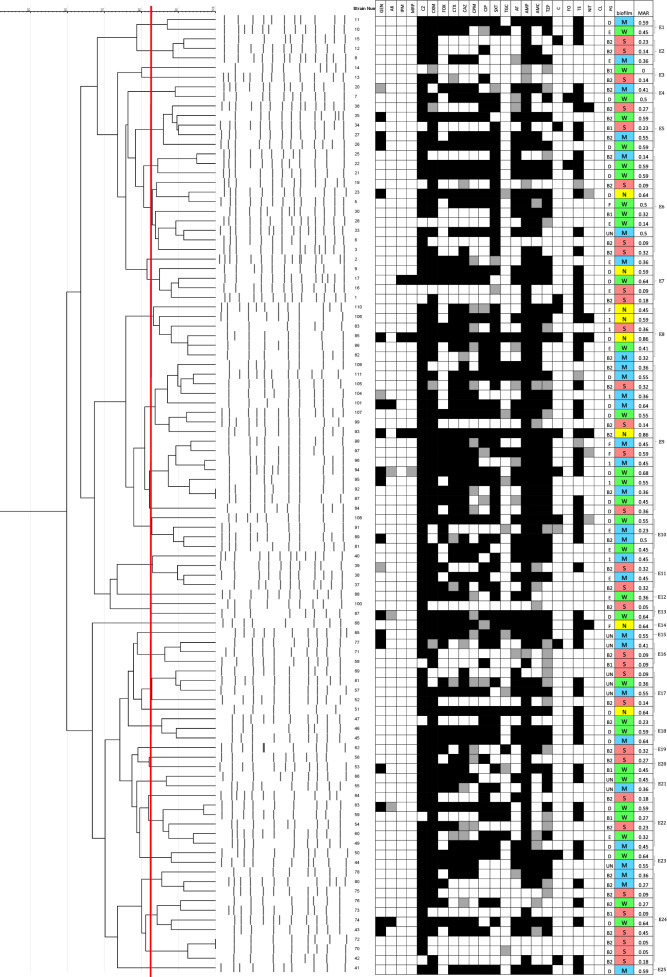
ERIC-PCR dendrogram created by GelJ^®^ software. The red dashed line indicates the 80% similarity cutoff. Clustering was performed using the UPGMA linkage and dice coefficient. The antibiotic resistance profile for each isolate is included, where black squares represent resistance, grey squares represent intermediate resistance, and white squares represent susceptibility. *PG* phylogenetic group. Biofilm formation categories are also included. *S* strong biofilm-forming isolate, *M* moderate biofilm-forming isolate, *W* weak biofilm-forming isolate, *N* non-biofilm-forming isolate

To evaluate the clonal relatedness and variability among the tested isolates, ERIC-PCR fingerprinting was used. Our findings revealed that the isolates possessed considerable genetic diversity. The number of resulting bands ranged between 5 to 12 bands per isolate, with band sizes between 100 to 2000 bp (Additional file 7: Figure S5). DNA fingerprint analysis using GelJ^®^ identified a total of 25 ERIC types which were designated as E1 to E25 (Fig. 6). Among these types, E9, E6, and E24 were the most prevalent, including 16, 11, and 10 isolates, respectively. On the other hand, E12, E14, E15, E19, and E25 were composed of only one isolate each.

Isolates 70 and 72 were both phylogroup B2 members, that had identical fingerprints (100% similarity), almost identical resistance patterns, and displayed strong biofilm formation. Isolates 87 and 92, belonging to phylogroups D and B2, respectively, also had identical fingerprints and very similar resistance patterns, and had weak and moderate biofilms, respectively. Some types had higher proportions of strong/moderate biofilm-forming isolates, such as type E24, where 8 out of 10 isolates belonging to this type had strong/moderate biofilms, but otherwise, the ERIC types possessed variable characteristics and no significant correlation to antimicrobial resistance, biofilm formation or virulence characteristics was detected.

## Discussion

This study aims to explore the phylogenetic grouping, antimicrobial resistance, biofilm formation capacity, and virulence profile of 100 clinical UPEC isolates collected from Tanta University Hospital, Egypt. Understanding these factors and the underlying correlations among them helps provide valuable epidemiological knowledge to guide the local empirical treatment of UTIs in Tanta, improve our grasp of the disease progression, and facilitate the creation of novel treatment approaches to prevent the persistence and recurrence of UPEC infections [55].

In the present study, 90% of the tested UPEC isolates were MDR/XDR. Regarding other regions in Egypt, Hassuna et al. found that 62% of UPEC isolated in Upper Egypt were MDR in 2020 [56], and a recent study published in 2022 by Mohamed et al. reported that 80% of UPEC were MDR in Alexandria governorate, Egypt [57]. These results reflect the increasing trend of AMR in Egypt, which is strongly linked to poor patient compliance, self-medication, and the availability of antibiotics as over-the-counter medications [58]. Globally, variable rates of multidrug resistance were reported in UPEC, for example, MDR rates of 62.25%, 73%, 64.9%, and 14.4% were reported in UPEC isolates from China, Iran, Nepal, and Hungary, respectively [3, 10, 18, 59].

The variability of resistance patterns in *E. coli* strains across different regions highlights the importance of current local information about UPEC resistance patterns to refine the antibiotic prescription process [60]. In the current study, the isolates had elevated resistance rates to β-lactams, SXT, and CIP. In other Egyptian governorates, UPEC isolated from patients in Minya city, the capital of Minya governorate, had the highest resistance rates against β-lactam antibiotics [61], while resistance rates for UPEC isolates from Alexandria city, the capital of Alexandria governorate, were highest against β-lactams, trimethoprim-sulfamethoxazole, and fluoroquinolones [62]. The high prevalence of resistance against these antibiotics is associated with their frequent administration for the management of UTIs in Egypt [57]. On the other hand, we observed that colistin, fosfomycin, amikacin, imipenem, meropenem, nitrofurantoin, chloramphenicol, and tigecycline had the highest efficacy with resistance rates ranging between 0 and 13%. In *E. coli* isolated from Mansoura hospitals, Egypt, El-baz et al. detected lower resistance rates against tigecycline (2%), and higher resistance rates against fosfomycin (4.67%) and colistin (7.33%) [11].

The elevated resistance observed in this study toward β-lactams may be attributed to β-lactamase activity [63]. Our results revealed that 67% of the isolates possessed at least one of the three tested ESBL genes (*bla*_TEM_, *bla*_SHV_, and *bla*_CTX-M_). Several studies reported that the dissemination of ESBL-producing *E. coli* has been steadily rising in Egypt. For instance, in a study published in 2019 by Shash et al., 38.8% of the tested UPEC isolates in Cairo, Egypt were ESBL producers [64]. El-shaer et al. in 2021, reported that 59.7% of *E. coli* from different sources were ESBL-producers in Mansoura, Egypt [65]. Globally, the spread of ESBL-producing *E. coli* varies greatly, with a prevalence of 15%, 18.3%, and 20.4% in Europe, North America, and Saudi Arabia, respectively [66, 67]. Higher rates were reported in Nepal, Sudan, and Libya, where 62.1%, 65%, and 67% of *E. coli* isolates produced ESBLs, respectively [59, 68, 69]. Of the three tested genes, the most prevalent ESBL gene in the current study was *bla*_CTX-M_, followed by *bla*_TEM_ and then *bla*_SHV_. A similar distribution was found in different regions in Egypt [61, 62]. Conversely, Hassuna et al. found that *bla*_TEM_ was the predominant ESBL gene in UPEC, followed by *bla*_CTX-M_, and then *bla*_SHV_ in the Minya governorate, Egypt [56]. We found that most of the ESBL-producing isolates showed the coexistence of more than one ESBL gene, which was reported previously in other Egyptian governorates [9, 61]. Furthermore, ESBL-positive isolates in the current study had significantly higher resistance rates to non-β-lactam antibiotics, such as GEN, CIP, and SXT. In this study, CIP-resistant isolates also showed significant co-resistance to other antibiotics. This co-resistance phenomenon represents a significant challenge for treatment and may be explained by the simultaneous carriage of ESBLs and other resistance genes in the same transposable genetic elements [61, 62, 70, 71]. Additionally, acquiring fluoroquinolone resistance can induce efflux mechanisms leading to cross-resistance to other antibiotics [72, 73].

Biofilms impart the embedded cells with up to 1000-fold higher resistance compared to their planktonic counterparts [74], which makes the treatment and eradication of biofilm-related infections extremely difficult [14, 75]. Our findings reveal that 92% of the isolates could form biofilms, with 60% classified as strong/moderate biofilm producers. Biofilm formation rates in UPEC have great global variation, and rates between 43.2% and 85% were previously reported [3, 13, 17, 18, 70]. As for different governorates in Egypt, EL-baz et al. reported biofilm formation in 89.3% of *E. coli* isolated from Mansoura hospitals in Dakahlia governorate, with 53.9% grouped as strong/moderate biofilm formers [11]. Similarly, strong/moderate biofilm formation was observed by 56.25% of *E. coli* in Minya University Hospital, Minya governorate [6].

Understanding how biofilm formation relates to AMR supplies valuable information for the development of preventive measures for biofilm-associated infections. Numerous studies found inconsistent results regarding the relationship between resistance and biofilm formation [13, 76, 77]. In the current study, a significant negative correlation was detected between antibiotic resistance and biofilm formation capacity. Upon investigation of this relationship for each of the tested antibiotics individually, we found a statistically significant negative relationship between biofilm formation and resistance to 17 antibiotics (Fig. 4). However, the absence of a significant relationship in the case of AK, FO, C, and CZ may be a result of the great contrast in resistance distribution to these antibiotics. This inverse correlation where the sensitive strains have a high ability to form biofilms may suggest that biofilm formation ensures the survival of the less resistant strains when exposed to antibiotics [20, 77].

The impaired antimicrobial diffusion due to biofilms means that embedded cells are exposed to sub-lethal concentrations of these agents, which promotes biofilm formation, this is called adaptive tolerance and occurs through metabolic or transcriptional adaptation without genetically acquired resistance [74, 78–80]. Because this tolerance is not expressed in the planktonic state, it cannot be detected by conventional AST, leading to a major difference in the susceptibility of biofilm-associated cells and planktonic cells of the same strain. Subsequently, standard susceptibility tests cannot be relied on to estimate the resistance of biofilm-mediated infections [20, 24]. In accordance with our observations, Poursina et al. found that strong biofilm producers of UPEC in Iran included significantly more non-MDR isolates [18]. The negative relationship between biofilm and resistance determinants indicates the “fitness cost” trade-off previously described in the literature, which suggests that acquiring resistance takes a toll on bacterial cells by reducing virulence, transmissibility, or growth rate [19, 79, 81, 82]. To that end, we observed that ESBL-possessing isolates had significantly weaker biofilms. Similar findings were previously reported in other bacterial species [83, 84]. The energy needed to express β-lactamases and carbapenemases was suggested as a cause for the diminished biofilm formation capacity [20], *bla*_TEM_ was also reported to impose a phenotypic cost in *E. coli* and *P. aeruginosa* leading to reduced biofilm formation [85].

A wide variety of virulence determinants possessed by UPEC strains contribute to their capacity to colonize the urinary tract and cause disease [7, 86]. We found that 65% of the isolates displayed iron chelation using the CAS assay, and that siderophore-producing isolates were significantly more resistant to β-lactams, chloramphenicol, and ciprofloxacin. In agreement with our findings, Asadi Karam et al. observed that CTX and CAZ resistance had a significant association with the presence of the siderophore genes *fyuA* and *iutA* in UPEC isolates in Iran [87]. Similar findings were also reported in *Enterococcus* [88]. This association can be explained by the presence of large plasmids which simultaneously carry virulence genes with antibiotic resistance genes, the selection of these plasmids by antibiotics concurrently selects for the associated virulence traits [6].

Hemolysin production facilitates UPEC invasion into renal epithelial cells and induces acute kidney damage [89]. Sixty-four percent of the tested isolates displayed hemolytic activity, which is higher than what was previously reported from different regions in Egypt. For instance, only 16.8% of the UPEC isolates demonstrated hemolysis in Cairo [90], while 42.2% of *E. coli* isolated in Minya hospitals were hemolytic [6]. Studies performed on UPEC in other countries also reported lower hemolysis rates of 41.5% and 32.3%, in Mexico and Nepal, respectively [70, 91]. We also observed that beta-hemolytic isolates were significantly more susceptible to cephalosporins, CIP, and GEN, which may be due to the reduced virulence as a cost of acquiring resistance, as reported previously [46, 73]. Hemolytic isolates were also significantly associated with biofilm formation, which agrees with previous reports stating that biofilm-forming UPEC had a higher rate of hemolysin production [92, 93]. Several studies reported that the hemolysin gene *hlyA* in UPEC is clustered on the same pathogenicity islands as the adhesin genes *pap*, and *sfa/foc,* the important role of these adhesins in biofilm formation may explain this relationship [17, 93]. Sixty percent of the isolates in the current study were serum-sensitive, while only 11% were serum-resistant, and 29% had intermediate resistance. The XDR isolates were serum-sensitive, and no statistical correlation between serum resistance and biofilm formation ability was found. Considering the ascending nature of UTIs, flagellar motility was suggested as a crucial virulence factor that gives UPEC cells a significant fitness advantage in the urinary tract [28, 94]. Although previous studies reported the importance of flagellar motility in the biofilm formation in *E. coli* [95, 96], we didn't find a significant association between motility and biofilm formation. Similar results were reported by Gajdács et al. in *P. aeruginosa* [97], while Nassar et al. reported a negative correlation between motility and biofilm formation in *P. aeruginosa* [98].

Monitoring the phylogenetic groups of *E. coli* is crucial to visualize the population structure, elucidating the relationship between different phylogroups and their virulence and resistance traits [11], as well as predicting disease prognosis and identifying emerging groups of bacteria [8, 56, 99, 100]. Studies on the phylogenetic distribution of UPEC in Tanta, Egypt using the modified Clermont scheme are scarce. Extraintestinal pathogenic *E. coli* strains typically belong to groups B2 and D [99]. In the present study, the majority of the tested isolates belonged to the phylogenetic group B2 (37%), and group D (25%). In agreement with our results, various studies observed that phylogroup B2 predominated in UPEC, followed by group D [8, 101–103]. In contrast, some studies found that phylogroup D is the most prevalent one [104, 105]. In other Egyptian governorates, such as Giza and Dakahlia, a similar phylogenetic distribution of UPEC was reported [106]. However, studies performed on isolates from University Hospitals of Mansoura and Minya cities in Egypt revealed that the predominant group in UPEC was group A, followed by group B2 and then D [107, 108]. On the other hand, a recent study found that phylogroup B2 prevailed in UPEC isolated in the Minya governorate, followed by group F [56]. These discrepancies may be due to variations in sample size, host genetic factors, and geographical distribution [8]. We demonstrated that, when compared to group D, group B2 isolates were associated with strong biofilm formation and beta hemolysis, and lower rates of ESBL production and antibiotic resistance. This is consistent with earlier research that found group B2 to be more virulent and group D to be more resistant, suggesting a trade-off between resistance and virulence [8, 10, 99].

## Conclusion

Our results provide comprehensive epidemiological data on the local resistance characteristics of UPEC in Tanta, the capital of the Gharbia governorate, Egypt, which contribute to the evidence-based refinement of the antibiotic prescription, to optimize the infection treatment and combat the antimicrobial resistance epidemic. We also demonstrate the alarming spread of multidrug resistance and the co-carriage of multiple ESBL genes, which are serious public health issues that require ongoing monitoring. An interesting inverse correlation between antibiotic resistance and biofilm formation was revealed, which stresses the importance of evaluating the majorly overlooked biofilm formation ability in clinical settings instead of solely depending on AST, as this may be an answer to the mystery of failure of antibiotic therapy and persistence of infections despite in vitro susceptibility. The potential fitness cost of important resistance genes and their negative effect on biofilm formation is also explored in this work. Further molecular investigations and in vivo studies can elucidate the role of biofilms in the resistance of UPEC to antibiotics.

### Supplementary Information

**Additional file 1: Table S1.** Demographic data of the patients.

**Additional file 2: Table S2.** MAR indices of the tested UPEC isolates (*n* = 100).

**Additional file 3: Figure S1.** Gel electrophoresis results of the multiplex PCR reaction for the detection of ESBL genes: *bla*_SHV_ (747 bp), *bla*_CTX-M_ (593 bp), and *bla*_TEM_ (445 bp). M; DNA ladder (bp).

**Additional file 4: Figure S2.** Representative photos of phenotypic virulence determination. Panel (**A**) represents hemolysis detection on blood agar, where 1, 2, and 3 display positive hemolysis demonstrated by the clear zones around the bacterial growth, while 4, 5, and 6 display negative results. Panel (**B**) represents siderophore detection on CAS agar plates, where 1, 2, and 3 display positive results, demonstrated by the golden yellow color around the bacterial growth, while 4 displays negative results.

**Additional file 5: Figure S3.** Gel electrophoresis results of the quadruplex PCR reaction used for phylogenetic grouping for the detection of the main four genes: *arpA* (400 bp), *chuA* (288 bp), *yjaA* (211 bp), and TspE4.C (152 bp). M; DNA ladder (bp).

**Additional file 6: Figure S4.** Gel electrophoresis results of the group E-specific PCR reaction. This reaction detects the gene *arpA* (301 bp), using the gene *trpA* (489 bp) as an internal reaction control. M; DNA ladder (bp).

**Additional file 7: Figure S5.** Gel electrophoresis results of the ERIC-PCR reaction. M; DNA ladder (bp).

## Data Availability

All data used and/or analyzed during this study are available in this manuscript and its Additional file data.

## Abbreviations

AMR: Antimicrobial resistance
ATCC: American type culture collection
CAS: Chrome azurol S
CLSI: Clinical Laboratory Standards Institute
CLED: Cystine lactose electrolyte-deficient
CLSM: Confocal laser scanning microscopy
CV: Crystal violet
DDST: Double disk diffusion synergy test
EPS: Extracellular polymeric substance
ESBL: Extended-spectrum β-lactamase
ERIC-PCR: Enterobacterial repetitive intergenic consensus-PCR
EUCAST: The European Committee on Antimicrobial Susceptibility Testing
HDTMA: Hexadecyltrimethylammonium bromide
LB: Luria bertani broth
LM: Light microscopy
MALDI-TOF\MS: Matrix-assisted laser desorption/ionization-time of flight mass spectrometry
MAR: Multiple drug resistance index
MDR: Multidrug resistance
OD: Optical density
PBS: Phosphate buffered saline
PCR: Polymerase chain reaction
SEM: Scanning electron microscopy
SDD: Sensitive dose-dependent
TSB: Tryptic soy broth
UPEC: Uropathogenic *Escherichia coli*
UPGMA: Unweighted pair group method with arithmetic mean
UTIs: Urinary tract infections
XDR: Extensively drug-resistant
