# Supplementary material for: Correlation between antimicrobial resistance, biofilm formation, and virulence determinants in uropathogenic Escherichia coli from Egyptian hospital
Source: Ann Clin Microbiol Antimicrob. 2024 Feb 24;23:20. doi: 10.1186/s12941-024-00679-2 (PMC10894499; doi:10.1186/s12941-024-00679-2)
Supplement: Supplementary file 1 — Additional file 1: Table S1. Demographic data of the patients. [file 12941_2024_679_MOESM1_ESM.docx]

**Supplementary Data**

**Table S1** Demographic data of the patients

| **Isolate code** | **Sex** | **Age** |
| --- | --- | --- |
| **EC1** | Female | 4 |
| **EC2** | Female | 29 |
| **EC3** | Female | 4 |
| **EC5** | Male | 72 |
| **EC6** | Male | 26 |
| **EC7** | Female | 47 |
| **EC8** | Female | 64 |
| **EC9** | Female | 66 |
| **EC10** | Female | 3 |
| **EC11** | Male | 72 |
| **EC12** | Female | 19 |
| **EC13** | Female | 26 |
| **EC14** | Female | 2 |
| **EC15** | Male | 75 |
| **EC16** | Female | 2 |
| **EC17** | Female | 40 |
| **EC19** | Female | 32 |
| **EC20** | Female | 23 |
| **EC21** | Female | 39 |
| **EC22** | Male | 70 |
| **EC23** | Female | 60 |
| **EC25** | Female | 70 |
| **EC26** | Male | 75 |
| **EC27** | Female | 31 |
| **EC28** | Female | 32 |
| **EC30** | Female | 2 |
| **EC33** | Female | 60 |
| **EC34** | Female | 20 |
| **EC35** | Female | 72 |
| **EC36** | Female | 65 |
| **EC37** | Female | 65 |
| **EC38** | Female | 23 |
| **EC39** | Female | 50 |
| **EC40** | Female | 4 |
| **EC41** | Female | 65 |
| **EC42** | Male | 65 |
| **EC43** | Female | 33 |
| **EC44** | Female | 15 |
| **EC45** | Male | 51 |
| **EC46** | Female | 72 |
| **EC47** | Female | 75 |
| **EC49** | Male | 42 |
| **EC50** | Female | 54 |
| **EC51** | Male | 72 |
| **EC52** | Female | 6 |
| **EC53** | Female | 3 |
| **EC54** | Female | 25 |
| **EC55** | Female | 5 |
| **EC56** | Female | 70 |
| **EC57** | Male | 47 |
| **EC58** | Female | 14 |
| **EC59** | Female | 22 |
| **EC60** | Female | 67 |
| **EC61** | Female | 7 |
| **EC62** | Female | 23 |
| **EC63** | Female | 39 |
| **EC64** | Male | 67 |
| **EC65** | Female | 9 |
| **EC66** | Female | 40 |
| **EC67** | Female | 55 |
| **EC68** | Female | 30 |
| **EC69** | Male | 19 |
| **EC70** | Female | 41 |
| **EC71** | Female | 6 |
| **EC72** | Female | 12 |
| **EC73** | Female | 2 |
| **EC74** | Female | 68 |
| **EC75** | Female | 33 |
| **EC76** | Female | 76 |
| **EC77** | Female | 30 |
| **EC78** | Male | 1 |
| **EC80** | Female | 61 |
| **EC81** | Female | 37 |
| **EC82** | Female | 33 |
| **EC83** | Female | 9 |
| **EC84** | Female | 52 |
| **EC85** | Female | 69 |
| **EC86** | Female | 54 |
| **EC87** | Female | 27 |
| **EC88** | Female | 32 |
| **EC89** | Female | 5 |
| **EC91** | Female | 6 |
| **EC92** | Female | 46 |
| **EC93** | Male | 78 |
| **EC94** | Male | 63 |
| **EC95** | Male | 75 |
| **EC96** | Female | 76 |
| **EC97** | Female | 34 |
| **EC98** | Female | 65 |
| **EC99** | Female | 42 |
| **EC100** | Female | 40 |
| **EC101** | Male | 70 |
| **EC104** | Female | 33 |
| **EC105** | Male | 38 |
| **EC106** | Female | 58 |
| **EC107** | Female | 70 |
| **EC108** | Female | 70 |
| **EC109** | Female | 30 |
| **EC110** | Female | 30 |
| **EC111** | Female | 29 |
