# Supplementary material for: Correlation between antimicrobial resistance, biofilm formation, and virulence determinants in uropathogenic Escherichia coli from Egyptian hospital
Source: Ann Clin Microbiol Antimicrob. 2024 Feb 24;23:20. doi: 10.1186/s12941-024-00679-2 (PMC10894499; doi:10.1186/s12941-024-00679-2)
Supplement: Supplementary file 2 — Additional file 2: Table S2. MAR indices of the tested UPEC isolates (n = 100). [file 12941_2024_679_MOESM2_ESM.docx]

**Supplementary Data**

**Table S2** MAR indices of the tested UPEC isolates (*n = 100*)

| **Isolate code** | **Number of antibiotics the isolate resists** | **MAR index*** |
| --- | --- | --- |
| **EC1** | 4 | 0.1818182 |
| **EC2** | 8 | 0.3636364 |
| **EC3** | 7 | 0.3181818 |
| **EC5** | 11 | 0.5 |
| **EC6** | 2 | 0.0909091 |
| **EC7** | 11 | 0.5 |
| **EC8** | 8 | 0.3636364 |
| **EC9** | 13 | 0.5909091 |
| **EC10** | 10 | 0.4545455 |
| **EC11** | 13 | 0.5909091 |
| **EC12** | 3 | 0.1363636 |
| **EC13** | 3 | 0.1363636 |
| **EC14** | 0 | 0 |
| **EC15** | 5 | 0.2272727 |
| **EC16** | 2 | 0.0909091 |
| **EC17** | 14 | 0.6363636 |
| **EC19** | 2 | 0.0909091 |
| **EC20** | 9 | 0.4090909 |
| **EC21** | 13 | 0.5909091 |
| **EC22** | 13 | 0.5909091 |
| **EC23** | 14 | 0.6363636 |
| **EC25** | 3 | 0.1363636 |
| **EC26** | 13 | 0.5909091 |
| **EC27** | 12 | 0.5454545 |
| **EC28** | 3 | 0.1363636 |
| **EC30** | 7 | 0.3181818 |
| **EC33** | 11 | 0.5 |
| **EC34** | 3 | 0.2272727 |
| **EC35** | 13 | 0.5909091 |
| **EC36** | 6 | 0.2727273 |
| **EC37** | 7 | 0.3181818 |
| **EC38** | 10 | 0.4545455 |
| **EC39** | 7 | 0.3181818 |
| **EC40** | 10 | 0.4545455 |
| **EC41** | 13 | 0.5909091 |
| **EC42** | 4 | 0.1818182 |
| **EC43** | 10 | 0.4545455 |
| **EC44** | 12 | 0.5454545 |
| **EC45** | 14 | 0.6363636 |
| **EC46** | 13 | 0.5909091 |
| **EC47** | 5 | 0.2272727 |
| **EC49** | 10 | 0.4545454 |
| **EC50** | 14 | 0.6363636 |
| **EC51** | 14 | 0.6363636 |
| **EC52** | 3 | 0.1363636 |
| **EC53** | 10 | 0.4545455 |
| **EC54** | 5 | 0.2272727 |
| **EC55** | 8 | 0.3636364 |
| **EC56** | 6 | 0.2727273 |
| **EC57** | 12 | 0.5454545 |
| **EC58** | 2 | 0.0909091 |
| **EC59** | 6 | 0.2727273 |
| **EC60** | 7 | 0.3181818 |
| **EC61** | 8 | 0.3636364 |
| **EC62** | 7 | 0.3181818 |
| **EC63** | 13 | 0.5909091 |
| **EC64** | 4 | 0.1818182 |
| **EC65** | 12 | 0.5454545 |
| **EC66** | 10 | 0.4545455 |
| **EC67** | 14 | 0.6363636 |
| **EC68** | 14 | 0.6363636 |
| **EC69** | 2 | 0.0909091 |
| **EC70** | 1 | 0.0454545 |
| **EC71** | 2 | 0.0909091 |
| **EC72** | 1 | 0.0454545 |
| **EC73** | 2 | 0.0909091 |
| **EC74** | 14 | 0.6363636 |
| **EC75** | 2 | 0.0909091 |
| **EC76** | 6 | 0.2727273 |
| **EC77** | 9 | 0.4090909 |
| **EC78** | 8 | 0.3636364 |
| **EC80** | 6 | 0.2727273 |
| **EC81** | 10 | 0.4545455 |
| **EC82** | 7 | 0.3181818 |
| **EC83** | 8 | 0.3636364 |
| **EC84** | 9 | 0.4090909 |
| **EC85** | 19 | 0.8636364 |
| **EC86** | 9 | 0.4090909 |
| **EC87** | 10 | 0.4545455 |
| **EC88** | 8 | 0.3636364 |
| **EC89** | 11 | 0.5 |
| **EC91** | 5 | 0.2272727 |
| **EC92** | 9 | 0.4090909 |
| **EC93** | 19 | 0.8636364 |
| **EC94** | 15 | 0.6818182 |
| **EC95** | 12 | 0.5454545 |
| **EC96** | 10 | 0.4545455 |
| **EC97** | 13 | 0.5909091 |
| **EC98** | 10 | 0.4545455 |
| **EC99** | 3 | 0.1363636 |
| **EC100** | 1 | 0.0454545 |
| **EC101** | 14 | 0.6363636 |
| **EC104** | 8 | 0.3636364 |
| **EC105** | 7 | 0.3181818 |
| **EC106** | 13 | 0.5909091 |
| **EC107** | 12 | 0.5454545 |
| **EC108** | 12 | 0.5454545 |
| **EC109** | 8 | 0.3636364 |
| **EC110** | 10 | 0.4545455 |
| **EC111** | 12 | 0.5454545 |

* MAR index is the Multiple antibiotic resistance index calculated by dividing the number of the antibiotics to which the isolate was resistant by the total number of the tested antibiotics (*n = 22*).
