# Supplementary material for: Correlation between antimicrobial resistance, biofilm formation, and virulence determinants in uropathogenic Escherichia coli from Egyptian hospital
Source: Ann Clin Microbiol Antimicrob. 2024 Feb 24;23:20. doi: 10.1186/s12941-024-00679-2 (PMC10894499; doi:10.1186/s12941-024-00679-2)
Supplement: Supplementary file 3 — Additional file 3: Figure S1. Gel electrophoresis results of the multiplex PCR reaction for the detection of ESBL genes: blaSHV (747 bp), blaCTX-M (593 bp), and blaTEM (445 bp). M; DNA ladder (bp). [file 12941_2024_679_MOESM3_ESM.docx]

**Supplementary Data**

**Figure S1** Gel electrophoresis results of the multiplex PCR reaction for the detection of ESBL genes: *bla*_SHV_ (747 bp)*, bla*_CTX-M_ (593 bp), and *bla*_TEM_ (445 bp). M; DNA ladder (bp)
