# Supplementary material for: Correlation between antimicrobial resistance, biofilm formation, and virulence determinants in uropathogenic Escherichia coli from Egyptian hospital
Source: Ann Clin Microbiol Antimicrob. 2024 Feb 24;23:20. doi: 10.1186/s12941-024-00679-2 (PMC10894499; doi:10.1186/s12941-024-00679-2)
Supplement: Supplementary file 4 — Additional file 4: Figure S2. Representative photos of phenotypic virulence determination. Panel (A) represents hemolysis detection on blood agar, where 1, 2, and 3 display positive hemolysis demonstrated by the clear zones around the bacterial growth, while 4, 5, and 6 display negative results. Panel (B) represents siderophore detection on CAS agar plates, where 1, 2, and 3 display positive results, demonstrated by the golden yellow color around the bacterial growth, while 4 displays negative results. [file 12941_2024_679_MOESM4_ESM.docx]

**Supplementary Data**

**Figure S2** Representative photos of phenotypic virulence determination

Panel **(A)** represents hemolysis detection on blood agar, where 1, 2, and 3 display positive hemolysis demonstrated by the clear zones around the bacterial growth, while 4, 5, and 6 display negative results. Panel **(B)** represents siderophore detection on CAS agar plates, where 1, 2, and 3 display positive results, demonstrated by the golden yellow color around the bacterial growth, while 4 displays negative results.
