# Supplementary material for: Correlation between antimicrobial resistance, biofilm formation, and virulence determinants in uropathogenic Escherichia coli from Egyptian hospital
Source: Ann Clin Microbiol Antimicrob. 2024 Feb 24;23:20. doi: 10.1186/s12941-024-00679-2 (PMC10894499; doi:10.1186/s12941-024-00679-2)
Supplement: Supplementary file 5 — Additional file 5: Figure S3. Gel electrophoresis results of the quadruplex PCR reaction used for phylogenetic grouping for the detection of the main four genes: arpA (400 bp), chuA (288 bp), yjaA (211 bp), and TspE4.C (152 bp). M; DNA ladder (bp). [file 12941_2024_679_MOESM5_ESM.docx]

**Supplementary Data**

**Figure S3** Gel electrophoresis results of the quadruplex PCR reaction for the phylogenetic grouping for the detection of the main four genes: *arpA* (400 bp)*, chuA* (288 bp), *yjaA* (211 bp), and TspE4.C (152 bp). M; DNA ladder (bp)
