# Supplementary material for: Correlation between antimicrobial resistance, biofilm formation, and virulence determinants in uropathogenic Escherichia coli from Egyptian hospital
Source: Ann Clin Microbiol Antimicrob. 2024 Feb 24;23:20. doi: 10.1186/s12941-024-00679-2 (PMC10894499; doi:10.1186/s12941-024-00679-2)
Supplement: Supplementary file 6 — Additional file 6: Figure S4. Gel electrophoresis results of the group E-specific PCR reaction. This reaction detects the gene arpA (301 bp), using the gene trpA (489 bp) as an internal reaction control. M; DNA ladder (bp). [file 12941_2024_679_MOESM6_ESM.docx]

**Supplementary Data**

**Figure S4** Gel electrophoresis results of the group E- specific PCR reaction, this reaction detects the gene *arpA* (301 bp), using the gene *trpA* (489 bp) as an internal reaction control. M; DNA ladder (bp)
