# Supplementary material for: Correlation between antimicrobial resistance, biofilm formation, and virulence determinants in uropathogenic Escherichia coli from Egyptian hospital
Source: Ann Clin Microbiol Antimicrob. 2024 Feb 24;23:20. doi: 10.1186/s12941-024-00679-2 (PMC10894499; doi:10.1186/s12941-024-00679-2)
Supplement: Supplementary file 7 — Additional file 7: Figure S5. Gel electrophoresis results of the ERIC-PCR reaction. M; DNA ladder (bp). [file 12941_2024_679_MOESM7_ESM.docx]

**Supplementary Data**

**Figure S5** Gel electrophoresis results of the ERIC- PCR reaction. M; DNA ladder (bp)
